# Prevalence and Amounts of Common Ingredients Found in Energy Drinks and Shots

**DOI:** 10.3390/nu14020314

**Published:** 2022-01-13

**Authors:** Andrew R. Jagim, Patrick S. Harty, Abdelrahman R. Barakat, Jacob L. Erickson, Victoria Carvalho, Chinguun Khurelbaatar, Clayton L. Camic, Chad M. Kerksick

**Affiliations:** 1 Sports Medicine, Mayo Clinic Health System, Onalaska, WI 54650, USA; Barakat.Abdelrahman@mayo.edu (A.R.B.); erickson.jacob@mayo.edu (J.L.E.); victoria.rvalho@gmail.com (V.C.); 2 Exercise & Sport Science, University of Wisconsin—La Crosse, La Crosse, WI 54603, USA; khurelbaatar0706@uwlax.edu; 3 Energy Balance and Body Composition Laboratory, Department of Kinesiology & Sport Management, Texas Tech University, Lubbock, TX 79409, USA; patrick.harty@ttu.edu; 4 Kinesiology and Physical Education, Northern Illinois University, DeKalb, IL 60115, USA; ccamic1@niu.edu; 5 Exercise & Performance Nutrition Laboratory, Lindenwood University, St. Charles, MO 63301, USA; ckerksick@lindenwood.edu

**Keywords:** energy product, energy drink, caffeine, taurine, alertness, performance

## Abstract

Background: Energy drinks are one of the most popular packaged beverage products consumed within the United States (US). Energy drinks are considered a functional beverage, a category that also includes sports drinks and nutraceutical beverages. Purpose: The focus of the current study was to examine the nutrition fact panels of the top selling commercially available energy drink and energy shot products within the US to characterize common ingredient profiles to help establish a standard definition and ingredient profile of energy drinks and energy shots for consumers, health care practitioners, and researchers. Methods: The top 75 commercially available energy drinks and shots were identified and compiled from multiple commercial retail websites as of September 2021. For the purpose of this study, an energy drink must have met the following criteria: (A) marketed as an energy drink; (B) purported to improve energy, focus, or alertness; (C) not sold as a dietary supplement (no supplement fact panels); (D) manufactured as a pre-packaged and ready-to-drink beverage; and (E) contains at least three of (1) caffeine, (2) B-vitamins, (3) sugar, (4) taurine, (5) creatine, (6) quercetin, (7) guarana, (8) ginseng, (9) coenzyme Q10, or (10) branched chain amino acids. Energy shots must have met similar criteria to be included: (A) marketed as an energy shot; (B) purported to improve energy, focus, or alertness; (C) sold as a dietary supplement; (D) manufactured as a pre-packaged beverage with a small volume (<3.5 mL); and (E) contains at least three of the ingredients stated above. Results: Twenty energy shots and fifty-five energy drinks were included in this analysis. The number of ingredients per product (mean ± SD) was 18.2 ± 5.7, with 15 products containing proprietary blends with undisclosed ingredient amounts. The relative prevalence and average amounts of the top ingredients were as follows: caffeine (100%; 174.4 ± 81.1 mg), vitamin B6 (72%; 366.9 ± 648.1 percent daily value (%DV)), vitamin B3 (67%; 121.44 ± 69.9% DV), vitamin B12 (67%; 5244.5 ± 10,474.6% DV), vitamin B5 (37.3%; 113.6 ± 76.6% DV), and taurine (37.3%; amounts undisclosed). Conclusions: Our findings suggest a high prevalence of caffeine and B-vitamins in these energy products, with many of the formulations containing well above the recommended daily value of B-vitamins.

## 1. Introduction

Energy drinks are one of the most popular packaged beverage products consumed within the United States (US). These products are considered a functional beverage, a category that also includes sports drinks and nutraceutical beverages [1]. Recently, annual energy drink sales have experienced tremendous growth, with estimated sales amounting to USD 2.8 billion in 2015 and sales up to USD 12 billion in 2020 (bevindustry.com, accessed on 15 September 2021). Energy drinks are commonly marketed to consumers with purported benefits of increased energy and alertness; reduced sensations of fatigue; enhanced physical performance; increased metabolic activity; and a plethora of other physiological, metabolic, cognitive, and performance benefits [2]. These ready-to-drink beverages often come in serving sizes of 8–16 oz. with 1–2 servings commonly found in a pre-packaged can and typically contain a combination of caffeine, vitamins, amino acids, electrolytes, and herbal extracts. Zero-calorie or sugar-free versions of each drink are commonly available commercially [2]. Similarly, energy “shots” are another commonly consumed pre-packaged beverage offering a similar ingredient profile, albeit in condensed versions, often with serving sizes of 3 oz. Historically, energy drinks first emerged to the market within the US in the late 1990s. Initially marketed towards young, active adults, these drinks are now regularly consumed by adolescents, young and older adults [3], athletes [4], and military personnel [5], with consumption rates tending to be higher in males compared with females [3]. While the prevalence of energy drink consumption has increased steadily in young adults from 0.5% to 5.5% during the span of 2003–2016, this consumption rate is still far below that of coffee, another popular caffeinated beverage, in which over half of the US population regularly consumes on a daily basis (mean intake: 54 g per day (~2.5 cups)), making up a majority of their daily caffeine intake and equating to a mean caffeine intake of 233 mg per day [6]. Comparatively, the United States Food and Drug Administration (FDA) states that amounts up to 400–600 mg per day appear to be safe for consumption, without any evidence of adverse effects when consumed below this threshold, whereas rapid ingestion of 1200 mg of caffeine appears to the upper end of the threshold for toxic effects, such as seizures or cardiovascular complications [7].

With the high caffeine content in certain energy drinks, in addition to the combination of varied ingredients, there have been concerns regarding both the acute and long-term safety of consuming these products. A growing number of case reports and clinical trials identifying various adverse effects following acute ingestion of energy drinks, namely acute hemodynamic perturbations, disturbances in vascular function, and other cardiovascular abnormalities, particularly when consumed in excess, have been reported [8,9,10,11,12,13]. Moreover, on a population level, it has been demonstrated that caffeine-containing products (such as energy drinks and shots) are associated with a higher risk of severe adverse events compared with non-caffeine containing products [14]. Furthermore, there have been reports of regular energy drink consumption being associated with sleep disturbances [15], anxiety [16], and substance abuse issues [17,18]. However, more work is needed to identify the exact mechanisms and whether the high caffeine content; combination of (and potentially excessive concentration of) vitamins, amino acids, and herbal extracts; or all of the above are contributors to the safety concerns and potential for cardiovascular-related adverse events.

Collectively, the consumption rates and popularity of these beverages are on the rise, and therefore, it is important to continue examining the prevalence of use as well as the acute and long-term effects of regularly consuming these products. More importantly, it is imperative to first establish a definition and a common ingredient profile to help provide context for when these products are included in future research activities or in conversations regarding the development of any policies, regulations, and guidance for use. Therefore, the focus of the current study was to examine the nutrition fact panels of the top selling commercially available energy drink and energy shot products within the US to help establish a standard definition and ingredient profile of energy drinks and energy shots for consumers, health care practitioners, and researchers.

## 2. Materials and Methods

The top 75 commercially available energy drinks were identified and compiled from multiple commercial retail websites (www.amazon.com, www.walmart.com, www.kroger.com, www.target.com, accessed on 7 September 2021) as of September 2021. Independent sales data were not available, so the included energy drink products were identified after filtering the searched products using the “bestselling” criteria previously used by Jagim and colleagues [19]. For the purpose of this study, the product must have met the following criteria to be classified as an energy drink and included in the analysis: (A) marketed as an energy drink; (B) purported to improve energy, focus, or alertness; (C) not sold as a dietary supplement (defined as not containing a supplement fact label); (D) manufactured as a pre-packaged and ready-to-drink beverage; and (E) contained at least four of (1) caffeine, (2) B-vitamins, (3) sugar, (4) taurine, (5) quercetin, (6) guarana, (7) ginseng, (8) coenzyme Q10, (9) electrolytes, or (10) branched chain amino acids. Sparkling water beverages with added caffeine were excluded from the analysis. These criteria were used to create distinctions between energy drinks and other similar products that may be marketed as energy drinks such as teas, sparkling waters, pre-workout supplements, thermogenics, or other performance-enhancing dietary supplements. If multiple formulations and drink types were available from a single manufacturer, only a regular and sugar-free (or low calorie) version was selected to avoid overrepresentation of a certain brand (and ingredient profile) within the data. Energy shots must have met similar criteria to be included: (A) marketed as an energy shot; (B) purported to improve energy, focus, or alertness; (C) sold as a dietary supplement; (D) manufactured as a pre-packaged beverage with a small volume (<3 fl oz.); and (E) contained at least three of the ingredients stated above.

Four research staff members independently analyzed each nutrition (or supplement) facts label to compile a list of ingredients and corresponding amounts, if available, for each product. Special notation was used for ingredients that were listed on the product label as part of a propriety blend, and therefore, specific ingredient amounts were not available. Only ingredients included in the nutrition facts section of the labels were included in the analysis. Thus, ingredients listed as “other ingredients” such as preservatives, artificial flavoring agents, and food coloring were not included in the analysis. As the information used for analysis is publicly available, approval from an Institutional Review Board was not required.

Where appropriate, the percent daily value (%DV) was calculated, if not initially included on the nutrition fact label, which can be in reference to the recommended dietary allowance (RDA) or the adequate intake (AI). In these cases, the %DV values were calculated based on recommendations from the Food and Drug Administration (https://www.fda.gov/food/new-nutrition-facts-label/daily-value-new-nutrition-and-supplement-facts-labels, accessed on 27 September 2021) using an adult male as the reference. These values included vitamin B3 = 16 mg; vitamin B6 = 1.7 mg; vitamin B12 = 2.4 mcg; vitamin B5 = 5 mg; potassium = 3400 mg; magnesium = 420 mg; and calcium = 1300 mg. Additionally, the following values were used to calculate the percentage of ingredients that were listed at amounts above the tolerable upper intake limit (UL) if known: vitamin B6 = 100 mg/day; vitamin B3 = 35 mg/day (https://www.ncbi.nlm.nih.gov/books/NBK56068/table/summarytables.t7/?report=objectonly, accessed on 27 September 2021).

### Statistical Analysis

All data are presented as means ± SD. Basic descriptive and frequency analysis was performed for the included ingredients across each product to create a common energy drink or energy shot ingredient profile. Means and standard deviations were reported for each ingredient explicitly listed on the nutrition or supplement fact label, where applicable. Frequency analysis was used to determine the relative prevalence of listed and unlisted ingredients within the dataset as well as the prevalence of ingredients that were found above the %DV or tolerable upper intake limit.

## 3. Results

### 3.1. Ingredient Frewuency and Totals 

Twenty energy shots and fifty-five energy drinks were included in this analysis. The number of ingredients per product (mean ± SD) was 18.2 ± 5.7, with 15 products containing proprietary blends with undisclosed ingredient amounts. One out of fifteen (6.67%) of the products containing proprietary blends was an energy drink, while fourteen of the fifteen products (93.33%) were energy shots. The relative prevalence and average amounts of the top ingredients were as follows: caffeine (100%; 174.4 ± 81.1 mg), vitamin B6 (72%; 367 ± 648 percent daily value (%DV)), vitamin B3 (67%; 12.41 ± 69.9% DV), vitamin B12 (67%; 5245 ± 10,475% DV), vitamin B5 (37.3%; 113.6 ± 76.6% DV), and taurine (37.3%; amounts undisclosed). 

### 3.2. Specific Ingredients

**Figure 1:**
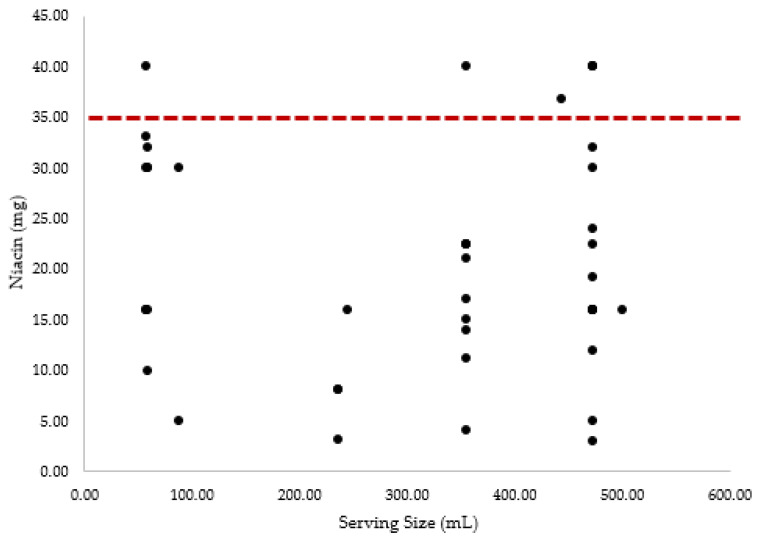
Niacin (vitamin B3) amounts plotted against serving size. Dashed line denotes UL for niacin (35 mg).

Table 1 provides the overall prevalence (%) of specific ingredients included in the 75 product formulations examined by this study. The prevalence for both undisclosed and listed amounts as well as the mean ± SD of the listed quantities per serving size were also calculated. These values only reflect products that listed ingredients in amounts larger than 0 mg or 0% DV. Of note, six products contained more niacin in a single serving than the UL (35 mg/day). Five of the six (83.33%) above the UL for niacin were energy drinks, while one out of six (16.67%) was an energy shot. Figure 1 provides a visual representation of the ingredient amounts for niacin, plotted against the serving size for each energy drink that contained the ingredient. The UL is also noted to highlight the number of products which contained amounts of niacin that were above the UL.

Table 2 provides the prevalence (%) of specific ingredients found in formulations from each product class. The prevalence for both undisclosed and listed amounts as well as the mean ± SD of the listed quantities per serving size were also calculated. These values only reflect products that listed ingredients in amounts larger than 0 mg or 0% DV.

## 4. Discussion

The primary aim of the current study was to examine the most prevalent ingredients found in commercially available energy drinks and energy shots to establish an operational description and common ingredient profile of these popular functional beverages and energy shots. The main findings of the current study indicate that all of the energy drinks and energy shots contain caffeine, while the majority of them also contain B-vitamins, sugar, taurine, ginseng, tyrosine, L-carnitine, and electrolytes. It is no surprise that caffeine is an ingredient found in all of the energy drinks and shots examined, as the physiological benefits of caffeine, namely its stimulatory effects, are in direct alignment with the purported benefits and marketing focus of these beverages. Previous research has found that caffeine ingestion alone can reduce sensations of fatigue while providing concomitant increases in feelings of energy and alertness and enhance mood [20]. Additionally, caffeine has previously been found to provide a variety of performance benefits for strength, anaerobic, and aerobic activities [20,21]. Although exhibiting somewhat of a similar ingredient profile as multi-ingredient pre-workout supplements, energy drinks and shots appear to contain more B vitamins (especially energy shots) and smaller amounts of beta-alanine, citrulline, creatine, and branched chain amino acids (BCAAs) compared with pre-workout supplements [19]. Furthermore, the caffeine content in pre-workout supplements appears to be higher, with mean values of 254.0 ± 79.5 mg compared with the 158.8 ± 73.8 and 217.1 ± 86.5 mg observed in the current study for energy drinks and shots, respectively. However, the consumption of caffeine (likely derived from any source) may exert ergogenic benefits when consumed prior to exercise and ingested at levels of 3 mg/kg of bodyweight or higher [20]. Therefore, each product could theoretically be used as an ergogenic aid, if caffeine content was above the ergogenic threshold. However, based on the average body weight of adult US males and females at 91 kg and 78 kg, the average amount of caffeine in energy drinks would equate to 1.7 and 2.0 mg/kg, respectively, which is below the ergogenic threshold. An interesting observation from the current analysis was the escalation of caffeine content found in energy drinks over the past 20 years. Red Bull was one of the original energy drinks that emerged in the US market in the late 1990s and contains 80 mg of caffeine per serving (250 mL), equating to 0.32 mg of caffeine per mL. Currently, BANG^©^ and Reign^©^ represent two of the more popular brands of energy drinks within the US, both of which contain 300 mg per serving (16 oz (473 mL)), representing 0.63 mg of caffeine per mL, which is 97% more caffeine content per mL compared with Red Bull.

Regarding the other prevalent ingredients, each one has been found to exert varying degrees of performance or cognitive benefits, with favorable safety profiles. For example, taurine is an abundant, semi-conditional amino acid with unique biochemical properties that has purported wide-ranging effects as a neurotransmitter with links to hormone function and energy metabolism [22]. Specifically, taurine is proposed to have both anti-inflammatory and antioxidant properties [23] with neuro-protective effects and may provide benefits for the treatment of various disorders such as heart failure, diabetes, and cystic fibrosis [22]. Despite the prevalence of taurine in energy drinks and other beverages, there are limited data on its effectiveness on metabolism when examined in isolation. Although taurine is present in mitochondria and plays a role in mitochondrial function, acute taurine supplementation of 1.0 g per dose has shown no effects on aerobic capacity, maximal heart rate, time-to-exhaustion, handgrip strength, vertical jump performance, or cognitive function [24]. Increases in fat oxidation, however, have been demonstrated during a 90 min cycling time trial with 1.7 g of taurine supplementation administered one hour prior to exercise [25]. It is also important to note that, while taurine supplementation appears to be generally safe at the examined doses, no tolerable upper limits on intake have been established [22]. Furthermore, the majority of the energy drinks included in the current study did not provide specific information regarding the dose of taurine included in the beverage and rather listed it as part of a proprietary blend. Ginseng is one of the most popular herbal medicinal products due to its potential widespread effects on fatigue, immunity, cardiovascular diseases, physical performance, as well as cognitive and sexual function [26,27]. Regarding safety, it appears that ginseng supplementation is considered generally safe but may be linked to relatively minor adverse events (i.e., headache, sleep issues, and gastrointestinal disorders). Reports of severe adverse events with ginseng are rare and few drug interactions (e.g., warfarin and alcohol) have been established [27]. Tyrosine is a non-essential amino acid and serves as a precursor to various catecholamines (e.g., epinephrine, nor-epinephrine, and dopamine). The availability of tyrosine in the brain has been suggested to influence the synthesis of these catecholamines and to improve their release in response to physical stress [28]. Specifically, it has been demonstrated that supplementation with tyrosine attenuates a decline in cognitive function during physical stressors such as cold exposure and sleep deprivation [29,30]. Although the limited research conducted on tyrosine supplementation indicates that adverse effects are extremely rare, the influence of long-term doses on safety have not been determined [28]. L-carnitine is an amino acid-like nutrient that stimulates the transport of long-chain fatty acids into the mitochondrial matrix. Based on this mechanism, it has widely been proposed that L-carnitine supplementation may increase lipid oxidation by mitochondria, thereby potentially enhancing the utilization of fat as a fuel source during exercise [31]. It has also been suggested that L-carnitine may serve as a protective mechanism from muscle damage and exercise stress by accumulating in capillary endothelial cells, by subsequently improving oxygen delivery to active muscles, by attenuating muscular hypoxia, by reducing tissue impairment, and by aiding in overall recovery [32,33]. From a safety standpoint, Rubin et al. [32] found that supplementation with L-carnitine L-tartrate (3 g·d^−1^ for three weeks) had no effect on markers of liver or renal function, minerals, electrolytes, and complete blood count compared with a placebo. Despite these potential ergogenic effects and general safety in healthy individuals, L-carnitine-related metabolites (i.e., acylcarnitines and trimethylamine-N-oxide) may be associated with increased cardio-metabolic risks in patients with diabetes [34]. Similar to taurine, no specific information regarding the common amount of L-carnitine included in the energy drinks from the current study was available. As such, there is no way to determine if sufficient amounts of these ingredients are present to elicit ergogenic value. Sugar was another common ingredient among the energy drinks examined in the current analysis. Several energy drink manufacturers tend to sell regular (sugar-containing) or low and/or zero-calorie versions of the same drink, with low or no sugar. Depending on the situation, the added carbohydrate source may serve as a key energy substrate, thereby potentially exerting ergogenic value during exercise and sporting activities. However, evidence is accumulating that indicates excess consumption of added sugars, namely in sedentary individuals, may be associated with negative health outcomes, such as obesity, metabolic disorders, and cardiovascular disease [35].

Regarding the safety of energy drinks, it is unlikely that the caffeine content alone is solely responsible for the potential for adverse events or safety concerns, considering the average caffeine amount in the energy drinks examined was 174 ± 81 mg. This amount of caffeine is similar to that of ~1.5 cups of coffee, which has not only been shown to be safe but also may confer a plethora of health benefits when combined with the polyphenols and antioxidant compounds found in brewed coffee [36]. Despite low risks when consumed in moderation (<400 mg), caffeine in excess (>600 mg) may result in various adverse events such as headaches, irritability, increases in heart rate, increases in blood pressure, gastrointestinal distress, and nausea [37]. Specifically, the threshold for adverse events appears to be dose-dependent in a linear fashion and may occur when caffeine ingestion exceeds amounts of 600 mg/day [38] or 9 mg/kg of bodyweight [39], although body size, genetics, and habitual intake may be influential factors [20]. This is approximately the amount of caffeine in 6 cups of brewed coffee or 3–4 servings of caffeine containing energy drinks, based on the findings from the current study. It is worth mentioning that caffeine is generally non-addictive, but tolerance may develop over time [38]. Therefore, over time, caffeine intake may need to be increased to continually achieve the same degree of desired effects, which may lead to excess consumption of caffeine-containing beverages. On the contrary, abrupt stoppage of caffeine in regular consumers can result in withdrawal symptoms, of which headaches, tiredness, difficulty concentrating, nausea, muscle pain, and irritability are commonly reported [40]. Collectively, these issues may promote routine and potentially excessive consumption of energy drinks over time, which could exacerbate any underlying safety concerns or health risks. Therefore, indiscriminate and excessive use of energy drinks and energy shots may increase the likelihood for adverse events and harmful side effects [2], which could be said for many functional beverages or dietary supplements. Moreover, recent findings suggest that certain genetic factors may influence how caffeine is metabolized, subsequently influencing its performance enhancing potential and the physiological responses post-consumption [20,41,42]. It is also important to note that the type of caffeine found in energy drinks is different to that which is extracted from coffee beans, such is the source found in brewed coffee. Caffeine sources in energy drinks are commonly derived from guarana, green tea extract, yerba mate, or synthetically derived, all of which may yield variations in pharmacokinetic profiles or how they are metabolized within the body. Previous work has also noted discrepancies in the caffeine content of energy drinks, compared with the amounts listed in the label [43], again warranting caution when consuming multiple caffeine-containing beverages throughout the day.

It may be that the mixed ingredient combination of B-vitamins, various herbal extracts, and amino acids collectively or in combination with caffeine may contribute to the potential for some of the adverse events and cardiovascular disturbances that have been previously reported [9]. As an example, Fletcher et al. [9] reported higher corrected QT intervals during an electrocardiogram (ECG) and higher systolic blood pressure values following consumption of an energy drink, when compared with a caffeine-matched control. These findings suggest that the combination of the added ingredients may somehow augment the sympathomimetic effects of caffeine. Regardless, more work is needed to examine the short and long-term effects of energy drink consumption in order to better understand the risk to benefit assessment of these beverages. Specifically, the comparison of energy drinks to other caffeine-matched beverages, such as coffee, tea, or caffeine anhydrous, is important to determine if the caffeine amount in energy drinks is the primary risk factor or if it is the combination of additive ingredients. Until then, it is not possible to discern primary causality regarding the risks of energy drinks consumption based on any one ingredient and the subsequent potential for adverse events.

An interesting finding from the current study is the amounts of ingredients that found above the %DV and, in some cases, above the UL established by the FDA. Specifically, niacin (vitamin B3) and vitamin B12, were found to be at levels ~115% and 1151% DV for energy drinks and ~142% and ~14,795% DV for energy shots. For niacin, this equates to 57.5% and 71% of the UL, and six of the products were found to contain more niacin in a single serving than the UL (35 mg/day). There is no established UL for vitamin B12, indicating that there is not currently sufficient evidence to identify a specific threshold at which adverse events may occur. B vitamins play an essential role in supporting many bodily functions including cell reproducibility, vision, cognitive function, digestion, metabolism, appetite regulation, cardiovascular health, and immune function as they serve as intermediates and enzymatic co-factors for several metabolic pathways [44]. However, excessive consumption of B-complex vitamins may result in adverse events, particularly if a person has any impairment in renal or liver function as it could potentially lead to a buildup of these vitamins. Common adverse events of excess B-complex vitamin consumption tend to include skin rashes, gastrointestinal distress, insomnia, and tingling sensations in rare cases [45].

A major challenge with research in this area is the robust variability in ingredients and dosages. For example, the range of vitamin B12 was 30–40,000% DV, which makes it challenging when making policy decisions about, determining the safety of, and examining the potential physiological effects of energy drink consumption. Additionally, little to no information is provided regarding any specific contextual information or sourcing of the ingredients on most labels. For example, it is unknown if the vitamin A found in these beverages is sourced from retinol or its synthetic analogues carotenoids. Furthermore, within the functional beverage industry, the lines are becoming increasingly blurred regarding distinctions between sodas, sparking water, sports drinks, and energy drinks. For example, multiple beverages predominantly classified as sparkling waters (based on prior marketing and beverage options) are now infusing their beverages with caffeine and other additives such as electrolytes, vitamins, minerals, etc. with marketing claims of “increased energy”, or other energy-centric and health-focused claims, which is somewhat misaligned from the original marketing focus and purported benefits of such beverages. Regardless, it is generally recommended that individuals with pre-existing cardiovascular, metabolic, hepatorenal, and neurologic diseases should avoid energy drinks and shots unless otherwise advised by a physician. Furthermore, it is recommended that consumers remain aware of serving sizes and refrain from consuming high volumes of energy drinks.

## 5. Conclusions

The present investigation established a standard definition of energy drinks and energy shots while identifying a common ingredient profile of each respective beverage. Our analysis indicates that all the energy drinks and energy shots included in the current study contain caffeine, while a majority of them also contain B-vitamins, sugar, taurine, ginseng, tyrosine, L-carnitine, and electrolytes. It is worth noting that many of the B-vitamin amounts included in several of the energy drinks and energy shots were found to be present in amounts well above the recommended daily value. Consumers should be cognizant of the ingredient profiles of energy products when making purchasing decisions, particularly when regularly consuming these products or when ingesting more than one serving of a given product at a time. Consumers and practitioners should pay close attention to the amounts of ingredients that may be prone to ‘mega-dosing’ such as niacin before consuming or recommending such beverages. Additionally, individuals who are caffeine naïve or smaller in stature (i.e., adolescents) should be cautious of energy drinks with a higher caffeine content to avoid the risk of adverse events. The findings of the current study help to establish a definition and summary of common ingredients, which can help future research when examining the specific health and performance effects of consuming these functional beverages, both in the short-term and after regular consumption over longer periods of time. Furthermore, these findings may help to guide future policy decisions regarding how these beverages are regulated and viewed regarding the potential health effects.

## Figures and Tables

**Table 1:** Prevalence and quantities of ingredients included in the bestselling energy drinks and energy shots (*n* = 75) per serving size.

Ingredient	Overall Prevalence (%)	Prevalence in Undisclosed Quantity (%)	Prevalence in Listed Quantity (%)	Mean ± SD Listed Quantity	Range
Caffeine (mg)	100	0	100	174 ± 81	45; 400
Vitamin B6 (%DV)	72.0	0	72	367 ± 648	17.7; 2353
Sodium (mg)	70.7	0	70.7	120 ± 118	2; 530
Niacin (%DV)	66.7	0	66.7	121 ± 70	18.8; 250
Vitamin B12 (%DV)	66.7	0	66.7	5245 ± 10,475	18.8; 41,666.7
Sugars (g)	45.3	0	45.3	19.9 ± 18.2	1; 63
Vitamin B5 (%DV)	37.3	0	37.3	114 ± 77	20; 400
Taurine	37.3	37.3	0	N/A	N/A
Potassium (mg)	34.7	0	34.7	148 ± 197	13; 830
Ginseng	30.7	30.7	0	N/A	N/A
Guarana	25.3	25.3	0	N/A	N/A
Vitamin C (%DV)	22.7	0	22.7	59.8 ± 48.7	3; 190
Tyrosine	22.7	22.7	0	N/A	N/A
Calcium (mg)	17.3	0	17.3	128 ± 175	5; 520
L-Theanine	17.3	17.3	0	N/A	N/A
Carnitine	16.0	16.0	0	N/A	N/A
Magnesium (mg)	12.0	0	12.0	25.4 ± 23.4	3.5; 74
Vitamin B2 (%DV)	8.0	0	8.0	133 ± 81	40; 260
Vitamin A (%DV)	6.7	0	6.7	78.6 ± 86.8	10; 220
Folate (mcg)	6.7	0	6.7	258 ± 194	40; 400
Vitamin D (%DV)	2.7	0	2.7	35.0 ± 21.2	20; 50
Choline (mg)	2.7	0	2.7	267 ± 330	33; 500
Vitamin B1 (%DV)	1.3	0	1.3	25.0	N/A

**Table 2:** Product class-specific prevalence and quantities of ingredients included in the bestselling energy drinks and energy shots per serving size.

	Energy Drinks (*n* = 55)			Energy Shots (*n* = 20)		
Ingredient	Prevalence in Product Class (% out of 55)	Mean ± SD Listed Quantity	Range	Prevalence in Product Class (% out of 20)	Mean ± SD Listed Quantity	Range
Caffeine (mg)	100	159 ± 74	45; 300	100	217 ± 87	45; 400
Vitamin B6 (%DV)	74.5	165 ± 199	17.7; 1250	65.0	1004 ± 1069	23.5; 2353
Sodium (mg)	76.4	143.1 ± 120.7	10; 530	55.0	30.5 ± 32.6	2; 100
Niacin (%DV)	70.9	115 ± 70	18.8; 250	55.0	143 ± 70	25; 250
Vitamin B12 (%DV)	63.6	1151 ± 4020	18.8; 20,833.3	75.0	14,796 ± 14,323	50; 41,666.7
Sugars (g)	52.7	22.0 ± 18.9	1; 63	25.0	7.6 ± 4.6	3; 15
Vitamin B5 (%DV)	47.3	111 ± 78	20; 400	10.0	150 ± 71	100; 200
Vitamin C (%DV)	25.5	63.6 ± 49.2	6; 190	15.0	41.7 ± 51.4	3; 100
Calcium (mg)	18.2	160 ± 189	5; 520	15.0	19.0 ± 17.8	5; 39
Magnesium (mg)	12.7	26.1 ± 24.6	4; 74	10.0	22.8 ± 27.2	3.5; 42

Ingredient prevalence represents the proportion of each ingredient per product class.

## Data Availability

Data available upon request.
